# Late positive slow waves as markers of chunking during encoding

**DOI:** 10.3389/fpsyg.2015.01032

**Published:** 2015-07-28

**Authors:** Ana M. L. Nogueira, Orlando F. A. Bueno, Gilberto M. Manzano, André F. Kohn, Sabine Pompéia

**Affiliations:** ^1^Departamento de Psicobiologia, Universidade Federal de São Paulo (UNIFESP)São Paulo, Brazil; ^2^Departamento de Neurologia/Neurocirurgia, Universidade Federal de São Paulo (UNIFESP)São Paulo, Brazil; ^3^Laboratório de Engenharia Biomédica, Departamento de Engenharia de Telecomunicações e Controle (PTC), Escola Politécnica da Universidade de São PauloSão Paulo, Brazil

**Keywords:** memory, distinctiveness, chunking, binding, clustering, event-related potentials

## Abstract

Electrophysiological markers of chunking of words during encoding have mostly been shown in studies that present pairs of related stimuli. In these cases it is difficult to disentangle cognitive processes that reflect distinctiveness (i.e., conspicuous items because they are related), perceived association between related items and unified representations of various items, or chunking. Here, we propose a paradigm that enables the determination of a separate Event-related Potential (ERP) marker of these cognitive processes using sequentially related word triads. Twenty-three young healthy individuals viewed 80 15-word lists composed of unrelated items except for the three words in the middle serial positions (triads), which could be either unrelated (control list), related perceptually, phonetically or semantically. ERP amplitudes were measured at encoding of each one of the words in the triads. We analyzed two latency intervals (350–400 and 400–800 ms) at midline locations. Behaviorally, we observed a progressive facilitation in the immediate free recall of the words in the triads depending on the relations between their items (control < perceptual < phonetic < semantic), but only semantically related items were recalled as chunks. P300-like deflections were observed for perceptually deviant stimuli. A reduction of amplitude of a component akin to the N400 was found for words that were phonetically and semantically associated with prior items and therefore were not associated to chunking. Positive slow wave (PSW) amplitudes increased as successive phonetically and semantically related items were presented, but they were observed earlier and were more prominent at Fz for semantic associates. PSWs at Fz and Cz also correlated with recall of semantic word chunks. This confirms prior claims that PSWs at Fz are potential markers of chunking which, in the proposed paradigm, were modulated differently from the detection of deviant stimuli and of relations between stimuli.

## Introduction

An important issue in memory research concerns the nature of encoding processes that make memory traces more easily accessible. Various mechanisms have been identified that increment future recall, but their electrophysiological markers are either unclear or difficult to dissociate from each other. Here we propose a paradigm that enables the behavioral and electrophysiological study of chunking during memory encoding. Chunking, also called binding, clustering, or grouping of information, reflects a compacted, optimized unitized representation of stimuli based on their abstract regularities and mnemonic redundancies and is known to lead to better recall (see Bor and Seth, 2012; Jaswal, 2012; Mathy and Feldman, 2012). This paradigm was designed to show that chunking can be dissociated from other cognitive processes that occur during encoding and that are also related to recall: distinctiveness and the detection of associations between items.

To better understand how these cognitive processes contribute to enhance memory it is useful to consider Fabiani and Donchin's (1995) model. This model predicts that the probability that an item will be retrieved at test depends on the extent to which memory is updated and organized during encoding. When the information is predictable, the processing of new information is minimized, whereas unexpected stimuli, detected primarily by bottom-up perceptual processes, elicit more allocation of mental resources (Bar, 2007), which in turn involve more conceptual processing. Thus, during the encoding phase of this model, items that stand out (distinctiveness) lead to the formation or restructuring/updating of their memory traces and this can lead to better recall (Otten and Donchin, 2000; Kelly and Nairne, 2001; Hunt, 2006).

The rehearsal phase of Fabiani and Donchin's (1995) model relates to elaborative strategies to organize memories by linking items together. Previous experiences and context can lead to the formation of retrieval paths for the to-be-remembered items due to strategic/goal-oriented and/or automatic/perceptual processes (Gobet et al., 2001). One example is the detection of associations or similarity between items which make them easier to recall (see Sederberg et al., 2010); another is the formation of connections between the to-be-remembered items into smaller meaningful units (see Tulving and Patkau, 1962; Cowan and Chen, 2009; Oberauer, 2010), or chunking (see Bor and Seth, 2012; Jaswal, 2012; Mathy and Feldman, 2012), which can occur based on semantic, phonetic or perceptual characteristics (Tulving and Pearlstone, 1966). The final phase of this model reflects cognitive processes that occur during retrieval (test phase) and which use the memory restructuring and elaborative strategies developed at earlier phases as retrieval tools (Fabiani and Donchin, 1995).

One way of obtaining objective measures of physiological processes that take place during encoding is to investigate Event-Related Potential (ERP) components that are associated to them (Fabiani and Donchin, 1995). ERPs are voltage fluctuations in the electroencephalogram, that are induced within the brain and are time-locked to some definable event, such as the presentation of a word (Rugg, 1995). They provide a direct, non-invasive measure of the temporal course of the voltage changes that are sensitive to manipulations of the cognitive context within which the eliciting stimuli are embedded (for a review, see Rugg, 1995). Additionally, during encoding, ERPs allow the online investigation of the functioning of working memory (Monfort and Pouthas, 2003), which is involved in chunking stimuli (see Baddeley, 2012; Jaswal, 2012; Kazerounian and Grossberg, 2014). Despite the fact that some forms of chunking also occur automatically, there are suggestions that attention and prior knowledge, or long-term memory, can also have a role in binding information together (see Baddeley, 2012; Jaswal, 2012). Electrophysiological correlates of chunking are, however, scant in the literature. Differently, the P300 and the N400 are accepted markers of distinctiveness and relational processing, respectively, as will be explained below.

The P300 is classically observed in oddball paradigms in which participants see or hear a series of repetitive stimuli and must detect rare or deviant stimuli among them, which elicit this deflection (see Rugg, 1995). In the context of learning information such as word lists, however, the amplitude of the ERP P300 component, which is most prominent at centro-parietal sites, reflects the degree to which the model of the context must be changed or updated and, therefore, the extent of memory reorganization at encoding (for a review, see Donchin and Coles, 1988). The consequences of mismatches during the early processing levels, which are directly related to P300 amplitudes (more mismatch leads to larger amplitudes), leave traces that lead to better episodic recall of individual items, mainly when rote memorization strategies are used at study (Karis et al., 1984; Fabiani and Donchin, 1995). This potential, therefore, has been considered a measure of primary *distinctiveness* (Schmidt, 1991; Hunt, 2006) which indicates data-driven processes (Fabiani and Donchin, 1995) related to context. Context in this case means, for example, how the participants consider that a particular stimulus relates to others in the same list (see Hunt, 2006). In contrast, when elaborative, top-down strategies are used during encoding of word lists, no recall advantage for distinctive words is found, and there is no correlation between the P300 amplitude and the recall probability of distinctive words (see Karis et al., 1984; Fabiani and Donchin, 1995; Fabiani, 2006). Instead, in these cases frontal positive slow waves (PSW) are related to recall, suggesting that elaborative cognitive processes are at play (see Kim et al., 2009).

Differently, the N400 is a negative-going potential that peaks centro-parietally over the scalp approximately 400 ms after stimulus onset (Lau et al., 2008; Kutas and Federmeier, 2011). Its amplitude is an inverse function of the relatedness between the target and prior context: the more negative or larger it is, the more it indicates the detection of a mismatch, while more positive or smaller N400 indicate the detection of similarity between stimuli and context (Kutas and Hillyard, 1984; Kutas and Federmeier, 2011). This deflection has been studied mainly in language processing; however, more negative N400 can be shown when not only semantic, but also phonological (e.g., Radeau et al., 1998; Grossi et al., 2001) and/or perceptual information is at odds with the context (see Kutas and Federmeier, 2011).

In general, the stimuli used in the above mentioned ERP studies that involved learning of sequences of items were manipulated in one of two ways: by the distinctiveness of isolated items, which induces the appearance of late frontal positivities that were connected to elaborative strategies and better recall (e.g., Karis et al., 1984; Fabiani et al., 1990; Fabiani and Donchin, 1995) or by the presentation of pairs of words, to observe the memory effects in associative processes (e.g., Weyerts et al., 1997; Kounios et al., 2001). In the latter case, it is impossible to determine which electrophysiological changes relate to the processing of which of the two stimuli in the pair. For example, when participants see two items at once, and realize that the second item is different from the first at a perceptual level, this would lead to the appearance of the P300 relative to the distinctiveness of the second stimulus. However, it would be impossible to know whether the P300 occurred due to the processing of the first or second word. Alternatively, subjects can perceive a semantic relation of the second item relative to the first, which would lead to a decrease in the negativity of the N400. Another possibility is that items can be chunked together after a relation of the second with the first item is perceived, which might result in a different ERP waveform. Thus, the resulting ERP changes could reflect many inseparable underlying cognitive processes.

The sequential presentation of items that can, in some way, be related, solves the abovementioned problem, but nevertheless has been used less often (see Kim et al., 2009, 2012). Kim et al. (2009, 2012) presented sequential words that could form related or unrelated pairs and showed that better recall was associated with the appearance of two deflections which they suggested improved memory formation: a less negative, or more positive, deflection in the 400 ms range, which they interpreted as a detection of relations between items, akin to what is described for the N400, and also a later PSW (frontal in the 2009 study, parietal in 2012 study) which they interpreted as related to formation of “bonds” between associated words. For this reason we decided to explore PSWs as possible ERP candidates of chunking.

However, using only two sequentially related items, as done by Kim et al. (2009, 2012), makes it difficult to electrophysiologically tease apart the cognitive processes that reflect the detection of an association between related items from chunking them together. This is so because the ERP changes related to both these processes would be observed only during the presentation of the second item of the pair, which can be associated and/or bound to the first. Behaviorally, it is also difficult to show that recall of two sequentially presented words constitutes a chunk and that they were not recalled better because they were individually encoded in a more efficient manner, or because of the perception that they were associated to each other. To understand this consider that chunking can be identified when participants recall items in the same order in which they were presented, even if free recall rather than serial recall is measured (see Tulving and Patkau, 1962). This type of learning in a unitized manner can occur as events are encoded consecutively and constitutes an essential skill for language acquisition and for remembering motor and spatial sequences (Kazerounian and Grossberg, 2014). When using two succedent chunkable items, if participants recall both words, there is a 50% chance that the words will be recalled in the same order as presented, making it difficult to establish whether the items where chunked into a single unit. The use of three sequentially related items (e.g., items A, B, and C) as proposed here is more adequate for this type of investigation because, if participants remember them all, the probability that recall will be in the same order as presented, by chance, is only 1 in 6 possible permutations of three items (e.g., ABC, BCA, CAB, etc.). Hence, if the recall exceeds this probability, it can be assumed that chunking occurred. Additionally, using three related and distinct items allows the assessment of the formation of smaller chunks of two items (e.g., AB, BC). Furthermore, comparing chunk sizes using different types of associations between items (e.g., semantic, phonetic, perceptual) can aid in the understanding of this chunking phenomenon because differences in the prior knowledge about the possible associations between stimuli should lead to differences in chunk size (see Cowan and Chen, 2009).

In sum, regarding ERP correlates of cognitive processing during encoding, it can be said that depending on the type of stimuli or context, different deflections are observed. Perceptually deviant stimuli elicit P300, detection of different types of relations between stimuli reduces the negativity of the N400 and elaborative, or organizational processes (possibly chunking), may lead to PSWs. The present study was designed to assess the encoding processes (distinctiveness, association between target items and chunking) that are related to successful retrieval in immediate free recall, and ERP correlates to these processes at encoding using three sequential related stimuli. Our aim was to provide a novel approach to study chunking mechanisms that can be dissociated from distinctiveness and detection of relations between items, which has not been undertaken in the literature.

To this end, we measured ERPs in a latency window that enables the study of P300, N400, and PSWs during memory encoding of sequentially presented word triads (e.g., see Andrade et al., 2003; Nogueira et al., 2006). These triads include items that are unrelated (e.g., car, pencil, apple), semantically related (e.g., milk, cheese, butter), phonetically (e.g., sea, fee, knee) or perceptually related (with a font type alteration in relation to the remaining items of the list, but without phonetic or semantic relations). These word triads were inserted in the middle serial positions of lists of 15 words that did not share these characteristics with the target-stimuli so that the related items differed from their context. Considering that immediate free recall of lists with more than 4 words can involve the use of working memory and long-term memory (see Rose and Craik, 2012; Kazerounian and Grossberg, 2014), we focused on the immediate free recall of the middle items (7th, 8th and 9th serial positions) in the list because they reflect the ability to store new information in long-term memory, regardless of the anchoring and rehearsal processes involved in primacy and recency (see Kahana, 1996; Nogueira et al., 2006). Electrophysiological markers of retrieval processes were not investigated here as our focus was on changes that occur during the encoding of information.

We hypothesized that there would be a progressive increase in free recall, in relation to triads with non-related words, for those with perceptually related items, followed by those with phonetically and semantically related words, as well as higher chunking for items that had more relational representations in long-term memory (i.e., higher for semantic followed by phonological relations; see Rose and Craik, 2012). Notably, the number of related words that could be bound together (three) in the present paradigm does not exceed the capacity limit of working memory (4 ± 1; see Cowan, 2010; Kazerounian and Grossberg, 2014) and can therefore be kept in this system while elaborative processing take place so as to link them with each other to form chunks. In terms of the ERP correlates, our hypotheses were as follows, regarding changes in ERP amplitudes: (a) that physically deviant words would elicit P300 components; (b) that the sequential presentation of semantically and phonetically related words would induce a decrease in the N400 negativity and an increase in positive slow waves (PSWs)—we believed that these effects would be larger for the semantic than the phonetic triads because of the nature of long-term memory, which benefits more from semantic associations (Rose and Craik, 2012); and (c) that it would be possible to show larger PSWs when chunk sizes were larger, especially at frontal sites (see Karis et al., 1984; Fabiani and Donchin, 1995; Fabiani, 2006; Kim et al., 2009). In order to show that the changes observed from the first to the last word in the triads were not secondary to a simple increase in to-be-remembered words, we also analyzed ERP alterations that occurred for the word that succeeded the last word in the triad (10th word of the list, when the pattern of unrelated words of the list was resumed). We expected that some distinctiveness (P300) could occur for this word after the related items of the triads had ceased. Also, the N400 of the 10th word of the lists with related triads could be more negative than that of the 9th word because it would be unrelated to the last word in the triad. We believed that if we were able to show these various deflections in different contexts (list types and serial positions within triads) that this paradigm would have the potential of indicating electrophysiological alterations that may further the understanding of mechanisms related to chunking, especially if they were found to be dissociable from markers of relational processes and distinctiveness.

We also considered whether our findings could be explained by computational models of chunking (see Kazerounian and Grossberg, 2014). The Adaptive Resonance Theory proposes that for chunking to occur, bottom-up signals related to each item interact with top-down expectations; these, in turn, select bottom-up features that are relevant to the task at hand and suppress those that are not (see Kazerounian and Grossberg, 2014). This reactivating cycle amplifies and synchronizes activity to certain groups of items and thus binds the attended features together into a coherent brain state (see Kazerounian and Grossberg, 2014). Thus, longer sequences of items generate larger recurrent inhibitory signals and top-down excitatory signals (see also Jaswal, 2012). Another model proposed by Kazerounian and Grossberg (2014) suggests that for chunking to take place, items presented sequentially are initially temporarily stored individually in an Item-and-Order fashion; this is followed by the activation of chunking networks that are sensitive to sequences of items of variable lengths, called Masking Fields. Item-and-Order, also called competitive queuing, involves an initial storage of items in a primacy gradient (i.e., activity related to each item in a sequence decreases progressively). Differently, Masking Fields, a special case of Item-and-Order, represent sequences of items (chunks), and not individual items. Larger Masking Fields, which correspond to chunks with more items (e.g., ABC), inhibit others which reflect storage of smaller sequences of items (e.g., AB, BC) and can also exert larger top-down excitatory signals than smaller chunks; thus, chunks encompassing more information prevail over chunks with less information. If PSWs are indeed markers of Masking Fields they should be largest when bigger chunks are recalled.

## Materials and methods

### Participants

This study involved 23 young, healthy adults [12 men, 2 left-handed, aged 25.2 ± 3.9 (mean ± SD) years, age range: 18–35] with more than 12 years of schooling, with normal or corrected vision, who had no history of neurological or psychiatric disorders, who were non-smokers and not under medication at the time of the study and for whom Portuguese was the native language. This study was approved by the Ethics Committee of the Universidade Federal de São Paulo, Brazil (project no. 0950-02), and all participants provided written informed consent.

### Stimuli

The stimuli were 1200 common Portuguese disyllabic and tri-syllabic concrete nouns with written frequency above 0.06/million in Portuguese (Berber, 2001). To compose lists, a pilot study was carried out in which the lists with 15 words were shown to roughly 50 undergraduates when they were attending classes. They were asked to indicate if the words were unknown, very distinct from the others or if there were any clear semantic or phonetic relations between the words. When any of these factors was pointed out by more than 20% of the individuals, the words were replaced and the list was submitted again to a new group of undergraduates using the same method. This was repeated until we obtained no more indications of relations. The relatedness of semantic and phonetic triads was checked in groups of 15 undergraduates who evaluated the associations in 100 mm-long horizontal lines (visual-analog scales). They were asked to mark on this line, which represented the full range of relatedness, the extent to which the stimuli were related, considering the left point of the scale as “no relation” and the opposite right point as “maximum possible relation”). Scores were measured as mm from the extreme left of the line. Only the triads with mean scores above 75 mm were included.

These selected words and triads were used to compose 80 15-word lists balanced according to the number of syllables and the frequency of the written words, which had no semantic or phonetic relations, except, in some cases, for the items located in the intermediary positions (serial positions 7, 8, and 9), referred to here as word triads.

**Figure 1:**
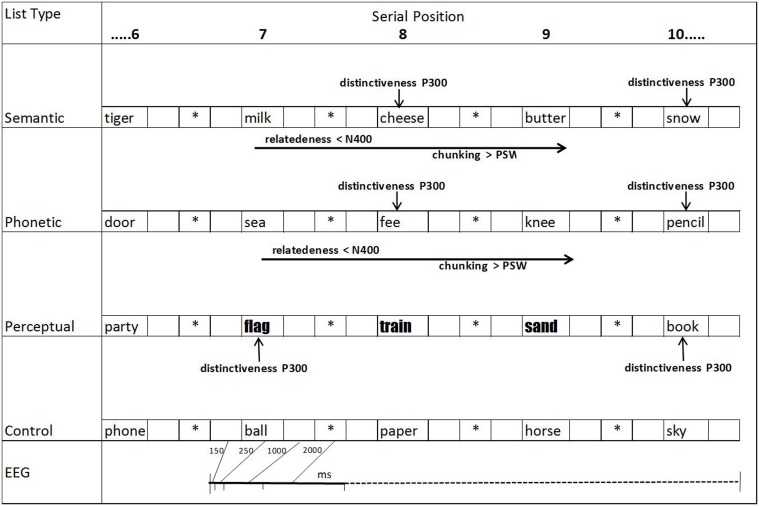
Schematic representation of the presentation of word lists, times when ERPs (EEG) were registered (150 ms before 7th word until 1000 ms after the 10th word; ^*^fixation points; blank spaces, blank screens) and expected deflections based on our hypotheses.

Four types of lists were used (20 of each kind), which will be called semantic, phonetic, perceptual, and control lists. The first two types of lists included word triads in the middle serial positions (positions 7, 8, and 9) that were semantically and phonetically related, respectively (see Figure 1). All the other words in these lists were unrelated to each other and to the words in the triads. The remaining 40 lists were composed entirely of unrelated words. Half of these were termed perceptual lists and included word triads written in a different font type; the remainder consisted of control lists with no manipulations of the triads (unrelated words in the same font as the other words in the list).

### Procedure

After placing Ag/AgCl electrodes on the scalp at frontal (Fz), central (Cz), and parietal (Pz) positions, according to the international 10–20 system (Jasper, 1958), volunteer saw a set of 80 lists of 15 words each. These lists were presented in a counterbalanced way at 4 weekly experimental sessions (20 lists per session). The words were presented sequentially on the computer screen in lowercase black Times New Roman font, point size 120, on a white background, except for the triads in the perceptual lists, which were formatted in the New Courier font, size 120. Following the procedure used in several studies (e.g., Fabiani and Donchin, 1995), each word was exposed on the screen for 250 ms, followed by a blank screen (1000 ms, during which ERPs were measured); an asterisk was then presented (2000 ms), followed by another blank screen (150 ms), the new word, and so forth (see Figure 1).

The participants were instructed to form a mental image of the meaning of each word to ensure that all words had been processed in an itemized fashion (strategic encoding) to avoid shallow processing that would have resulted in poor free-recall. They were told to try to memorize as many words as possible. Furthermore, this was undertaken so as to decrease the variability between participants in terms of the encoding strategies that were used. We did not direct participants to carry out associative encoding strategies (e.g., linking words together) as this could have altered the perception of context that varied between list types and, thus, the resulting P300 (i.e., elaborative encoding strategies remove the recall advantage for distinctive words; see Karis et al., 1984; Fabiani and Donchin, 1995; Fabiani, 2006). They were also instructed and trained to blink only during the presentation of the asterisk. At the end of each list, the subjects were asked to orally recall as many words as possible in any order (free recall) with no time limit. The score was the sum of the recalled words per serial position in each of the 4 types of lists in all the sessions (maximum of 20 words per serial position per list). Chunking measures are described in the Statistical analyses section.

### EEG recording

Only Fz, Cz, and Pz electrodes locations were investigated due to limitations of our equipment. These scalp locations were selected because they are essential to differentiate P300 and PSW (see Fabiani et al., 1986). The reference electrodes (M1 + M2) were positioned in the left and right mastoids, and a ground electrode was placed on the forehead, aligned with the Fz, Cz, and Pz. Ocular movements were monitored by electrodes placed in the right infraorbital and left supraorbital regions.

A programme developed in C language generated two channels of pulse synchronization for the Nihon Kohden, Neuropack, MEB 5508K equipment to record the ERPs (Cisi et al., 2000). The records were collected during the study phase in epochs beginning 150 ms before the onset of the 7th word and finishing 1000 ms after the presentation of the 10th word of each list. The signals were obtained using the “Gather Wave” setting of the Nihon Kohden equipment. Due to storage limitations of the equipment, the epochs were captured by synchronized pulses in two windows of examination (detailed in Cisi et al., 2000): (a) 150 ms before the onset of the 7th word until 1000 ms after the 8th word; (b) 150 ms before the onset of the 9th word until 1000 ms after the onset of the 10th word. ERP correlates to the 10th word were measured to investigate whether the change of context, after the related triads were presented, would elicit encoding changes. All the channels were amplified at a sensitivity of 50 μV/div and a bandwidth of 0.1–50 Hz. The impedances between the electrodes were maintained at or below 5 kΩ.

A program using Matlab software performed the following analyses: The correction of the vertical and horizontal scales to allow calibration in microvolts and milliseconds; the baseline was calculated over the 150 ms window preceding each word for every list and participant; the separation of the signals from each electrode (Fz, Cz, Pz) and list type (4 types: semantic phonetic, perceptual, and control); the separation of the relative potentials elicited by the 7th, 8th, 9th, and 10th words; the rejection of the potentials corresponding to the EEG derivations when the ocular movements associated with a word exceeded an absolute value of 200 μV; the rejection of signals in all of the EEG derivations, if the maximum value minus the minimum in the signal was greater than 100 μV; and the rejection of drifts higher than 75 μV. The program also calculated the percentage and mean values of all the accepted signals per subject, electrode, serial position, and list type. Participants with less than 60% valid signals were excluded. Finally, the grand averages, that represented the average of the accepted evoked potentials, were calculated. This study focused on the latency intervals related to the N400, P300, and PSW components. Thus, we considered the maximum amplitudes in the 350–450 ms range, which we expected would show N400 effects, and in the 400–800 ms windows, which should yield measures of P300 and PSWs. Following Fabiani et al. (1986), we used a classic mean amplitude functional approach to separate these latter deflections. P300 were considered those most prominent at Pz and Cz, when comparing deflections in the same serial positions among lists; PSWs had larger amplitudes at Fz in these comparisons. Changes in latencies were not analyzed because they could have varied according to the type of perceptual characteristics (see Kutas and Federmeier, 2011).

### Statistical analysis

The analyses with inferential purposes were performed using within-subjects, repeated measures analyses of variance (ANOVAs) followed by Tukey's Honest Significant Difference tests, which correct for multiple comparisons. The Greenhouse-Geisser correction was applied to correct for the violations of sphericity when necessary and was reported only if the results changed in comparison to the non-adjusted analyses. The factors and levels used in these analyses are detailed in the Results Section. The level of significance adopted was 5%. Data not reported below had *p*-values higher than 0.10. Effects sizes were indicated with eta squared in One-Way ANOVAS or partial eta squared (η^2^_*p*_) when more than one factor was investigated. Rules of thumb for considering minimum values to represent practical significant effects (Ferguson, 2009) were those higher than 0.04 (values higher than 0.25 indicated moderate effects and higher than 0.64, strong effect sizes).

Mean chunk size was determined following Tulving and Patkau (1962). Briefly, we determined for every list type the number of words recalled in chunks (one isolated word, two or three words recalled in the same input order) divided by the number of times any word was recalled. Hence, the minimum possible mean chunk size was zero (recall of none of the words) and the maximum was three (recall of all the three words in the same serial order as they were presented). Another chunking metric, recall of the three words of the triads in the same order as they were presented (here called three-word chunks) were also calculated. They could vary from 0 to 20 (number of lists per type of relations).

## Results

### Behavioral results

#### Recall by serial position

**Figure 2:**
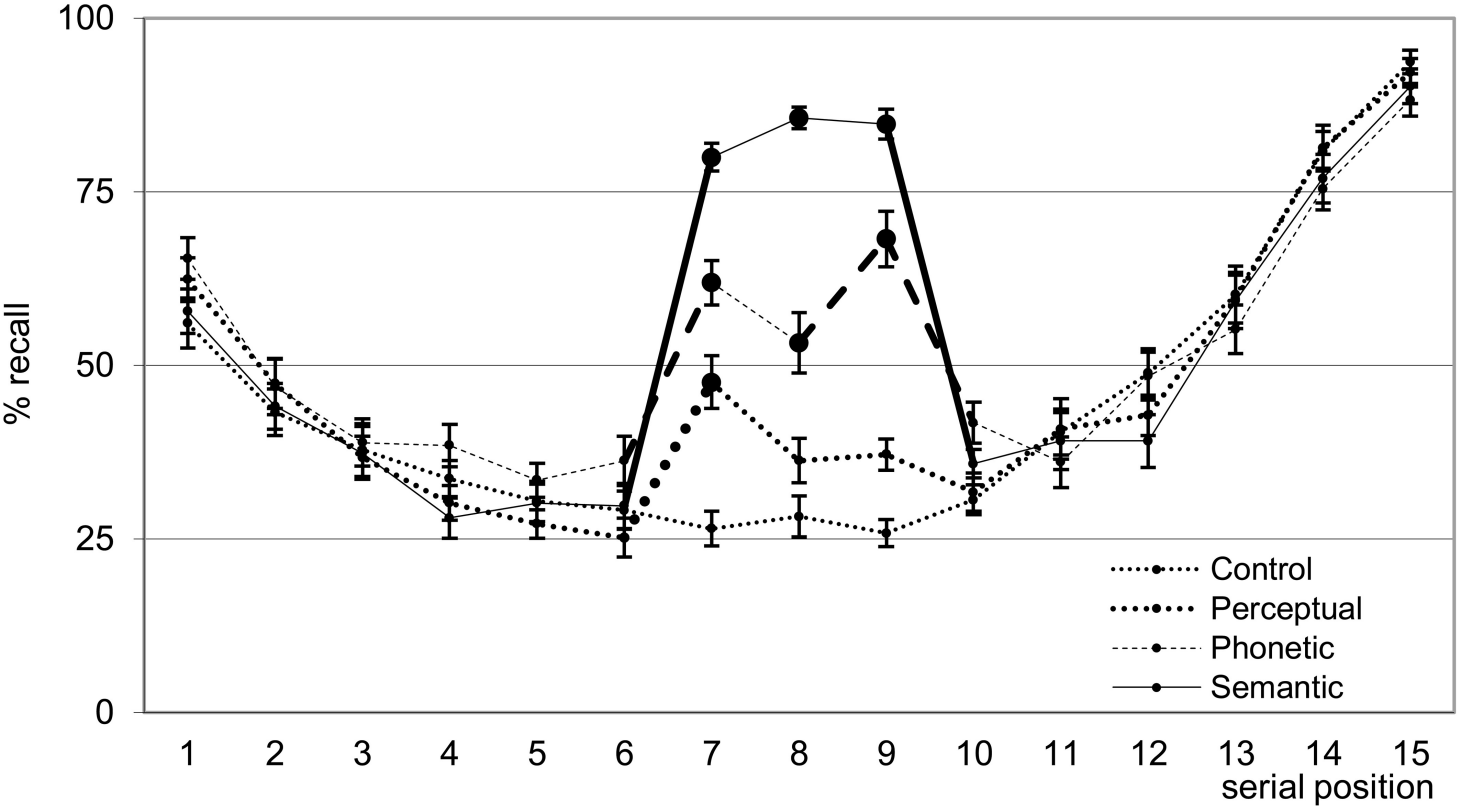
**Mean (±SE) of the percentage of words freely recalled according to serial position for the four list types**. Bold lines indicate difference between adjacent serial positions; large filled circles (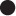) indicate difference from control lists. Bold segments indicate within list differences. See text for detailed statistical results.

Serial Position Analyses (Figure 2): Recall data of words in individual serial positions were analyzed by an ANOVA for repeated measures with the following factors: List type (4 levels: unrelated/control and words with semantic, phonetic, and perceptual relations), and the word position in the list (15 levels: Position 1–15). The maximum score per serial position was 20 because this was the number of lists per type of relation. The ANOVA showed effects of list type, position, and an interaction between these two factors [respectively, *F*_(3, 66)_ = 60.59, η^2^_*p*_ = 0.73; *F*_(14,308)_ = 88.57, η^2^_*p*_ = 0.80; *F*_(42,924)_ = 27.01, η^2^_*p*_ = 0.55; all *p* < 0.0001]. In relation to the effect of position, primacy and recency effects were clearly shown in all the lists. For the primacy effect, the 1st word was more often recalled than the 2nd and 3rd words (all *post-hoc p*-value contrasts < 0.001), which were recalled to the same extent. The recency effect was observed by an increase in recall after the 12th word (11th = 12th < 13th <14th < 15th; all *post-hoc p*-value contrasts < 0.001). List effect showed a grading in recall (semantic > phonetic > perceptual > control: all *post-hoc p*-value contrasts < 0.001).

The interaction occurred exclusively as a result of recall facilitation of the word triads (no differences within lists for words 1st–6th and 10th–15th; all *post-hoc p*-value contrasts > 0.10). Specifically regarding this interaction, within each list, recall of the three words in the triads were equivalent, except that the 8th word in the phonetic triad was less recalled than the 9th (all *post-hoc p*-value contrasts < 0.001). To illustrate that recall of words in the related triads was better than that relative to adjacent positions, we compared the recall of the 7th word with that of the 6th in all lists. The 6th word was used as a comparison because it also reflects immediate free recall without the influence of primacy and recency effects. The 7th word was better recalled than the 6th in the three types of related triads and was higher for the semantic, followed by the phonetic, perceptual and finally the unrelated lists (all *post-hoc p*-value contrasts < 0.001). Note that, in the case of the 7th word of the perceptual triad, this better recall than the 6th word indicates a distinctiveness effect. This explanation does not hold for the better recall of the 7th words in the semantic and phonetic triads because they were unrelated to, and thus undistinguishable from, previous words in the lists (see chunking analysis below and the discussion for a possible explanation of this memory facilitation). Comparisons among lists for the 8th and 9th words showed that recall was higher for semantic (higher than all), followed by phonetic (higher than perceptual and control), followed by perceptual and control words, which were non-distinguishable (all *post-hoc p*-value contrasts for significant differences < 0.001).

#### Chunking

Mean chunk sizes (mean ±SD) per list, which could vary from 0 to 3, were as follows: control (1.3 ± 0.2), perceptual (1.5 ± 0.3), phonetic (2.2 ± 0.5), semantic (2.8 ± 0.1). The mean chunk size data were analyzed with a within subject repeated-measures ANOVA with list type (4 levels) as factor. Chunk size differed between list types [*F*_(3, 66)_ = 158.10; *p* < 0.0001, η^2^ = 0.88] in a progressive way (control < all, perceptual < phonetic and semantic, phonetic < semantic; all *post-hoc p*-value contrasts < 0.001, except for a tendency of difference between control and perceptual triads: *p* = 0.06). Hence, semantically related words were not only recalled more often, but were usually recalled together in the same order as presented; words in the unrelated, control triads were recalled less often and singularly, and the other conditions led to intermediate values.

**Figure 3:**
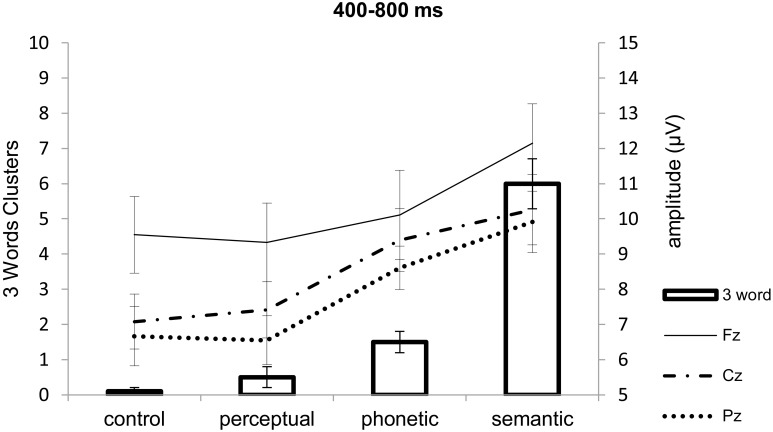
**Descriptive association [Mean (± SE)] of number of three-word chunks (recall of all three words in the triads in same order as presented) and positive slow wave amplitude (μV) at Fz, Cz, and Pz according to list type**. Maximum number of three-word chunks was 20 as there were 20 lists. See texts for correlations between three-word chunks and PSWs.

Three-word chunks, that is, recall of the three related words in the same order (which could vary from 0 to 20), occurred at a rate of 0.1 ± 0.3 (mean ± SD) for the control lists, 0.5 ± 1.1 for the perceptual, 1.5 ± 1.2 for the phonetic, and 6.0 ± 1.9 for the semantic lists (see Figure 3). A similar ANOVA as the one above [*F*_(3, 66)_ = 75.47; *p* < 0.0001, η^2^ = 0.77] revealed that more semantic three word chunks were recalled than all others, and that phonetic three-word chunks were better recalled compared to control ones (all *post-hoc p*-value contrasts < 0.02). In comparison to the occasions in which participants recalled all three words of the semantic triads in any order (control 0.7 ± 1.0; perceptual 1.9 ± 2.0; phonetic 6.3 ± 3.5; the semantic 14.3 ± 2.5), three-word semantic chunks represented a mean of 42.9% of recalls. This percentage is higher (*p* < 0.03, one-sided probability) than what would be expected by chance, considering 6 permutations of the three possible items to be recalled. These were thus considered “real” chunks. For control lists (5.4%), perceptual lists (15.6%), and phonetic lists (23.0%), these probabilities were equal to chance (all *p*-values > 0.11); therefore it cannot be said with certainty that chunking occurred in these cases.

### Electrophysiological results

**Figure 4:**
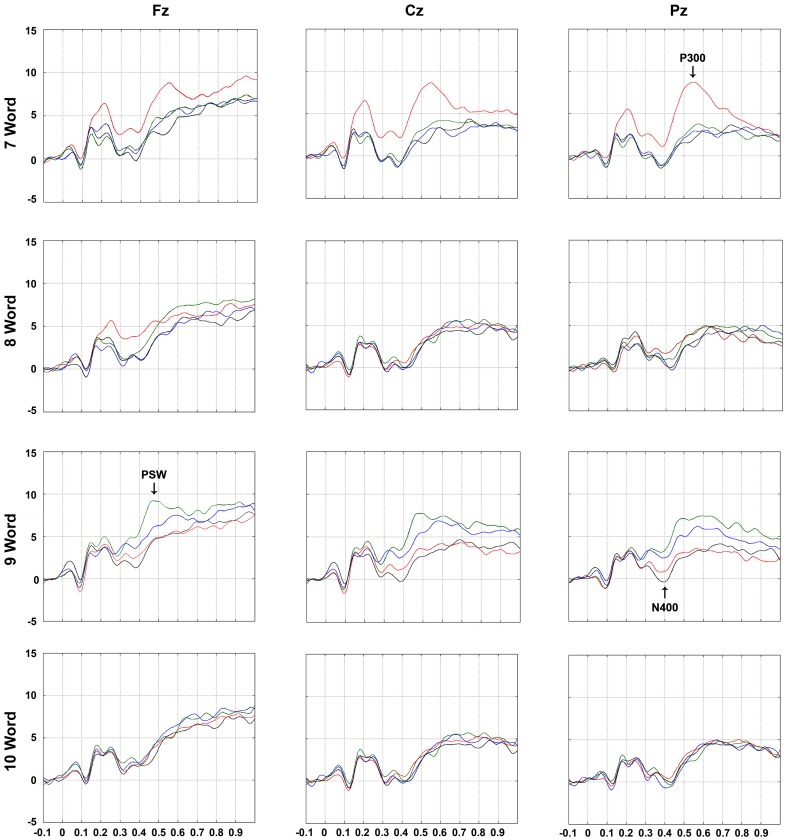
*****Grand Average*** ERPs elicited by word presentations according to electrode (Fz, Cz, and Pz), serial positions of interest (7th, 8th, 9th, and 10th), and list type (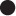 No relations: Control; 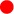 Perceptual relations; 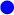 Phonetic relations; 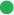 Semantic relations)**. Arrows illustrate studied deflections (P300, N400, and PSW = positive slow waves) where they reached maximum amplitude differences. Analysis windows were 350–450 ms range for N400 and 400–800 ms for P300 and PSWs. See Figure 1 for more details on serial positions.

Three subjects were excluded from the sample (one was left-handed) because they presented a valid signal contribution of less than 60%. The remaining participants had a mean of 75.0 ± 8.0% of valid signals. The grand average is shown in Figure 4.

The maximum amplitude means of each of the analyzed components was compared using an ANOVA for repeated measures with the following factors: Latency intervals of interest [350–450 ms (putative N400) and 400–800 ms (putative P300 and PSW); list type (unrelated, with semantic relation, phonetic relation, and perceptual relation); position of the word in the list (7, 8, 9, and 10); and electrode location (Fz, Cz, Pz)].

The amplitude analysis showed the effects of the latency intervals, electrode positions, list types, and serial positions [respectively, *F*_(1, 19)_ = 379.74, η^2^_*p*_ = 0.95; *F*_(2, 38)_ = 10.92, η^2^_*p*_ = 0.37; *F*_(3, 57)_ = 7.51, η^2^_*p*_ = 0.28; *F*_(3, 57)_ = 4.63, η^2^_*p*_ = 0.20, all *p*-values < 0.006], and an interaction among all these factors [*F*_(18, 342)_ = 1.86, *p* < 0.02, η^2^_*p*_ = 0.09].

**Figure 5:**
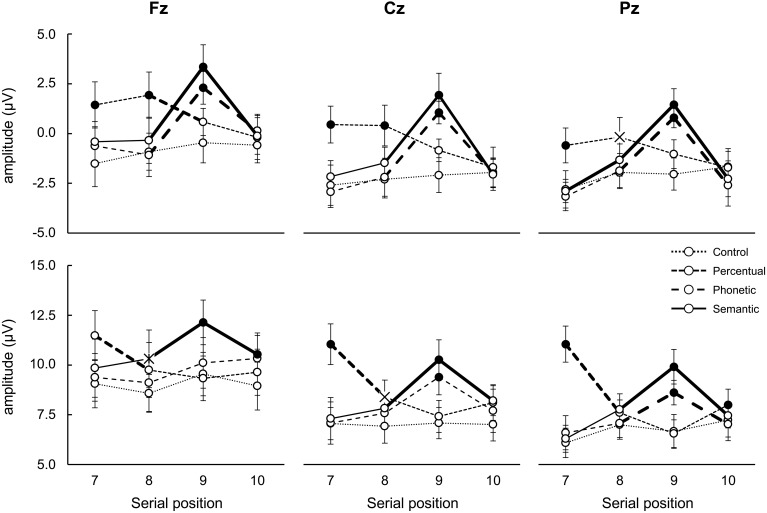
**Mean amplitude variation (μV; ±SE) relative to latency time-intervals 350–450 ms (upper panel, containing N400) and 400–800 ms (bottom panel, containing P300 and PSWs) according to electrode, serial position and list type**. Bold lines indicate difference between adjacent serial positions; filled circles (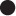) indicate difference between lists in the same serial position; X indicates different from control lists only. See text for detailed statistical results.

To facilitate the description of this interaction it is schematized below and described separately for both latency intervals (350–450 ms separately from 400 to 800 ms); in each latency interval we compared the deflections that occurred during the presentation of each word between lists (for words in the triads plus the 10th word in the list, when the unrelated pattern of the lists was resumed), and also among adjacent words in the same lists (e.g., 7th vs. 8th words, etc.) (Figure 5).

#### Latency intervals of 350–450 ms (putative N400)

##### Comparison among lists in each serial position

**7th word**: The amplitude after the onset of the 7th word of the perceptual triads was more positive than that of the 7th word in the other triads at all electrodes (all *post-hoc p*-value contrasts < 0.001), possibly due to a carry-over effect of a positive peak that occurred prior to the interval of interest, at 200 ms. This deflection may be associated with processing of visual features (e.g., see O'Donnell et al., 1997) or active inhibition of font characteristics, which were irrelevant for the encoding task (see Kotchoubey, 2006). This will not be discussed further because it reflects cognitive processes that this study was not designed to investigate. The reason for this is that it occurred only in this list type, and was not related to recall (note that this also happened for the 8th perceptual word which was not better recalled).
**8th word**: The amplitude after the onset of the 8th word of the perceptual lists was more positive at Fz and Cz than that of the remaining lists and at Pz only in relation to the control lists (all *post-hoc p*-value contrasts < 0.001); this result was also most likely due to an interference of a positive peak at 200 ms (see above);
**9th word**: There was a larger negativity for the items in the control and perceptual triads in relation to the semantic and phonetic triads irrespective of electrode (all *post-hoc p*-value contrasts < 0.001), indicating the perception of relatedness in the latter cases (reduction of N400). Semantic and phonetic signals did not differ.
**10th word**: No differences were found among triads.

##### Comparison among serial positions in the same list types

**7th vs. 8th serial positions**: There was a smaller negativity during the presentation of the 8th word in relation to the 7th at Pz in the semantic lists (*p* = 0.003) and a tendency of the same effect in the phonetic ones (*p* = 0.09). This indicates the detection of relations between words (N400);
**8th vs. 9th serial positions**: The amplitude after the onset of the 9th word was less negative than after the 8th one in the semantic and phonetic lists at all electrodes (all *post-hoc p*-value contrasts < 0.001), indicating detection of relations between words in both cases (N400). In the perceptual lists, the opposite occurred at Fz (*p* = 0.05), most likely due to the interference caused by the positive peak at 200 ms discussed above. Semantic and phonetic signals did not differ;
**9th vs. 10th serial positions**: The amplitude after the onset of the 9th word was less negative than that after the 10th item in the semantic and phonetic lists at all electrodes (all *post-hoc p*-value contrasts < 0.001), possibly due to the lack of relations of the 10th word with the last word in these triads. Deflections of the semantic and phonetic words did not differ.

#### Latency interval of 400–800 ms (putative peaks considered P300 and PSW)

##### Comparison among lists in each serial position

**7th word**: There was a higher positive peak triggered by the 7th word in the perceptual lists than in the other lists at all three electrodes (all *post-hoc p*-value contrasts < 0.001).
**8th word**: Compared with the same item in the control triads, the amplitude of the component observed after the onset of the 8th word was more positive in the perceptual lists at Cz (*post-hoc p* < 0.02). Together with the effect for the 7th word this indicated an effect of perceptual distinctiveness and, due to its scalp location, suggests it is a P300. The amplitude of the 8th semantic word at Fz was also more positive than that of the control list (all *post-hoc p*-value contrasts < 0.02). Because of scalp location, this indicates that it is a PSW;
**9th word**: Compared to the deflections elicited by the items in the perceptual and control triads, the component amplitude triggered by the 9th word was more positive in the semantic lists at the three electrodes and, for the item in the phonetic lists, at Cz and Pz (all *post-hoc p*-value contrasts < 0.001). Moreover, the wave elicited by the 9th word of the semantic lists was more positive than after the 9th word in the phonetic lists at Fz (*p* < 0.001). Because of scalp location, the effect for semantic words is interpretable as a PSWs, while those for phonetic ones indicate P300 waves;
**10th word**: The amplitudes elicited by the 10th word in the semantic lists were more positive at Fz than by the words in the perceptual and control lists (all *post-hoc p*-value contrasts < 0.001). This could have indicated a persistence of the PSWs described for the 8th and 9th words.

##### Comparison among serial position in the same list types

**7th vs. 8th serial position:** For the perceptual lists there was a higher positivity after the onset of the 7th in comparison to the 8th word at all electrodes (all *post-hoc p*-value contrasts < 0.001). For the semantic lists, the amplitude triggered by the 8th word was more positive than that elicited by the 7th word only at Pz (*p* < 0.01). This did not occur for the phonetic associates, nor for the control lists. These effects are compatible with P300 deflections because of scalp location;
**8th vs. 9th serial position:** The amplitude triggered by the 9th word was more positive than that elicited by the 8th word for the semantic lists at all electrodes and, for the phonetic words, at Cz and Pz only (all *post-hoc p*-value contrasts < 0.01);
**9th vs. 10th serial position:** A decrease in positivity was observed after the onset of the 10th word in the semantic lists in relation to the 9th word at all of the electrodes (all *post-hoc p*-value contrasts < 0.02); for phonetic lists this occurred at Cz and Pz (both *post-hoc p*-value contrasts < 0.001). In the perceptual lists, a higher positivity triggered by the 10th word in relation to the 9th at Pz was found (*p* < 0.02), suggesting the detection of change of font type back to that of the rest of the list, or a P300 due to its scalp location.

#### Exploratory analysis of the relation between chunking and frontal PSWs

**Figure 6:**
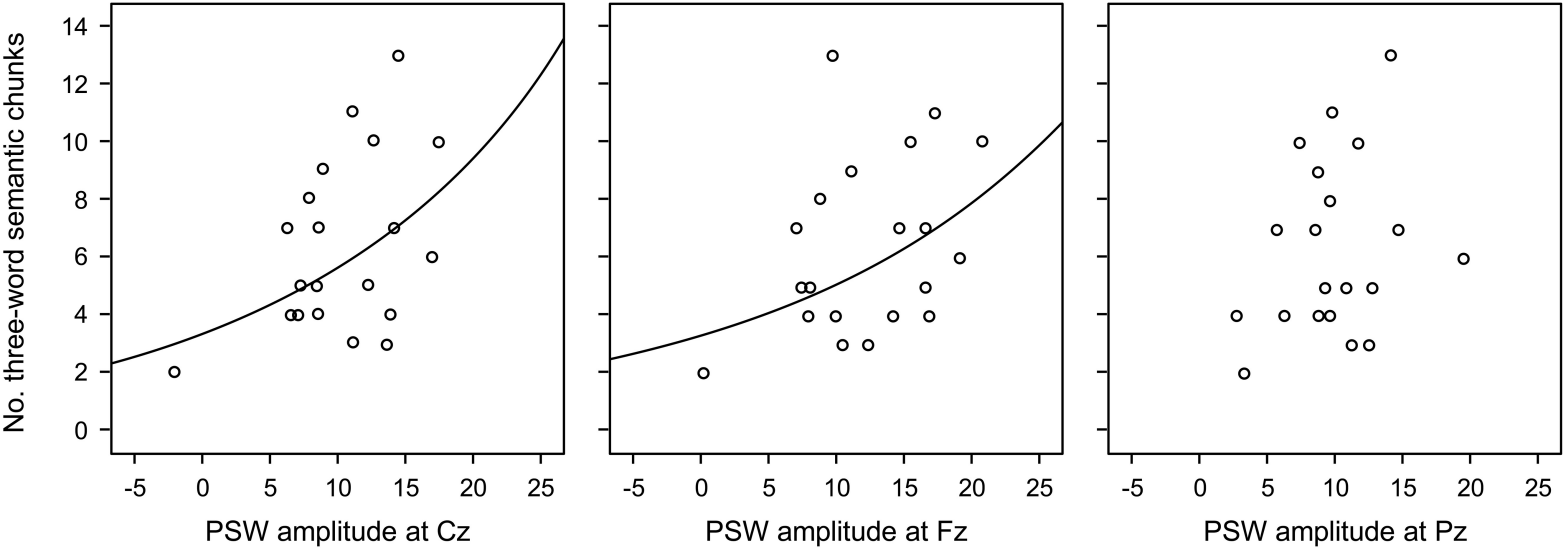
**Scatterplot of mean data per participant and fitted exponential regression line of Positive Slow Wave (PSW) amplitudes on the number of three-word semantic chunks (three words in the triads recalled in the same order as they were presented), per electrode**. The fit was significant at Fz (*R*^2^ = 0.20, *p* < 0.05) and Cz (*R*^2^ = 0.23, *p* < 0.04), but not Pz (*R*^2^ = 0.09, *p* = 0.20).

When all words in the semantic triad were recalled in the same order as presented, that is, as three-word chunks, which exceeded the probability of recalling items in this order by chance, the electrophysiological signals of this chunking process should have occurred most prominently when participants were exposed to the last related word in the triad (9th word). Supposing that PSWs could index chunking, as proposed in the literature, we compared the number of three-word chunks with the amplitudes of the PSWs during the presentation of this word. Figure 3 shows that: (a) the more positive the frontal PSW elicited by the 9th word in the triads, the larger the number of triads recalled in the same order as presented (three-word chunks); and (b) that this effect was dependent on list type. Because “real” chunking was only observed for the semantic triads, we investigate the relation between three-word chunks and amplitude of the PSWs at the three electrodes separately, using common regression models. Our data was better explained by exponential regressions, which were significant at Fz (*R*^2^ = 0.20, *p* < 0.05) and at Cz (*R*^2^ = 0.23, *p* < 0.04), but not at Pz (*R*^2^ = 0.09, *p* = 0.20) (see Figure 6). The fit was non significant when considering recall of three semantically related words *in any order* (Fz *R*^2^ = 0.006, *p* = 0.74; Cz *R*^2^ = 0.57, *p* = 0.57; Pz *R*^2^ = 0.04, *p* = 0.38). Following this, we carried out other exploratory analyses in the form of One-Way within-subject ANOVA on the amplitude of the PSWs measured during the presentation of the semantic lists, with electrode as factor [*F*_(2, 38)_ = 3.95, *p* < 0.03, η^2^ = 0.17; *post-hoc* contrasts Fz > Cz (tendency, *p* = 0.08) and Pz (*p* = 0.03)]. After controlling for the number of three-word semantic chunks (One-Way ANCOVA), the effect of electrode was no longer significant [*F*_(2, 36)_ = 0.68, *p* = 0.51, η^2^ = 0.03], suggesting again that chunking was related to the amplitude of the PSWs.

## Discussion

The behavioral results of the present study confirmed the initial hypotheses concerning differences in recall and chunking of words between the different types of word triads. The u-shaped immediate free-recall curve of the control lists reflected the classic primacy and recency effects (e.g., Kahana, 1996; Kazerounian and Grossberg, 2014) which were equivalent among lists except regarding the middle serial positions. Free recall of the semantic and phonetic lists was w-shaped, with an extra recall peak in the middle of the curve that corresponded to the related items, as previously shown (e.g., Capitani et al., 1992; Andrade et al., 2003; Nogueira et al., 2006; Kazerounian and Grossberg, 2014). Additionally, the distinctiveness of the change in the font type in the perceptual lists led to better recall of the first distinct item (words in the 7th serial positions) in comparison to the control condition, as found in many studies (e.g., Fabiani and Donchin, 1995). There was also a progressive increase in mean chunk size from the control through the perceptual, phonetic and semantic lists, which reflects the facilitation in recalling information that is already related in long-term memory (see Cowan and Chen, 2009; Baddeley, 2012; Jaswal, 2012). However, real chunking, that is, recall of the three related words in the same order as they were presented (three-word chunks; Tulving and Patkau, 1962) exceeded the probability of recall, considering all possible recall permutations of three items, only for semantic words. All behavioral and electrophysiological effects exceeded the minimum effect sizes to represent practical effects (see Ferguson, 2009).

Regarding the electrophysiological results relative to the triads, we also confirmed our hypotheses. We showed that three sequentially presented items related in different ways allowed the dissociation of three different ERP components that are associated to better recall, the P300, the N400 and PSWs. The first deflection was a component akin to the P300 for perceptually deviant stimuli. It occurred in the perceptual lists for the 7th (all electrodes) and 8th words (at Cz only) compared to the control lists. This deflection has been known to lead to memorization of individual items (e.g., Karis et al., 1984; Fabiani and Donchin, 1995; Otten and Donchin, 2000) and was associated to better recall of the 7th perceptual item. This peak progressively decreased from the first to the last word in the perceptual triads, possibly due to the decrease in distinctiveness (see Bar, 2007). Interestingly, compared to the deflection due to the 9th word in the perceptual list, there was a larger positivity at Pz once the font type reverted to that of the remainder of the list (10th word). This, together with the Cz effect for the 8th perceptual deviant word, supports the interpretation of this change as a P300 response to distinctiveness because of its electrode site, scalp distribution and functional significance (see Rugg, 1995). When participants saw the first deviant stimuli (7th word) in the perceptual list, the detection of a change in font was so marked as to be seen at all electrodes.

A similar P300-like effect was observed after the presentation of the 8th word in the semantic triads in relation to the 7th at Pz, suggesting a possible detection of distinctiveness, but in this case not a perceptual distinctiveness, but a change in the pattern of non-related items of the list. This confirms that stimuli can stand out in more than a perceptual way (Otten and Donchin, 2000). The lack of this type of effect for the second phonetically related words may have resulted from the weaker associations between these words in long-term (explicit and implicit/priming) memory (see Nelson et al., 1997; Rose and Craik, 2012). Additionally, semantic maintenance seems to be supported by brain systems that are distinct from the phonological ones (Shivde and Anderson, 2011). However, this difference between the effects for semantically and phonetically related items may have resulted from the encoding instructions, which did not focus on phonetic characteristics (see Morris et al., 1977; Nelson et al., 1987). This last hypothesis seems unlikely as a sole explanation for our effects because, despite the encoding instructions, phonetically related words were recalled at a much higher rate than the control and perceptually related words. This shows that participants benefited from the rhyming words when recalling items, which was shown as more positive deflection in this latency range at Cz and Pz later on, when comparing the 8th and the 9th phonetically related words.

The second component was measured in the latency window of 350–450 ms. After stimulus onset it indexed the detection of relatedness, which appeared as a reduction of the negativity of the deflections in the N400 range for phonetic (Grossi et al., 2001) and semantic items related to the prior words in the lists (Radeau et al., 1998; Kutas and Federmeier, 2011). This component followed the same pattern for both these types of lists, although behaviorally we found more chunking of semantic triads. Therefore, the N400 in the present set of data cannot be said to reflect chunking.

P300 and N400 changes induced by the 10th word, which succeeded the last word in the triads, showed that the observed effects were not secondary to an increase in the number of to-be-remembered items. There was an increase in the negativity of the N400 in the 10th word in the semantic and perceptual lists, likely due to the lack of relation with the last word in the triad. Also, a P300 was observed when the font changed back to the pattern of the list in the perceptual lists (distinctiveness). The ERP changes in other serial positions during free recall of unrelated word lists have been discussed by Azizian and Polich (2007) but will not be examined here as they do not relate to the aim of the present study.

The third type of detected component, which together with the P300 was observed in the latency window of 400–800 ms, were positive slow waves (PSWs). The presentation of the first item that was semantically related to the prior one (8th word in the semantic lists) elicited this component at Fz and was extended to all electrodes once the 9th word was presented. A similar deflection was observed for the phonetic words in comparison to the control items as well, but occurred only after the presentation of the last word in the triad (9th). In this case the deflection was observed at more posterior sites (Cz and Pz), and was less positive than that triggered by the last semantically related item at Fz. This observation indicates that, unlike the N400, the PSWs correspond to cognitive processing that was able to differentiate semantic chunking from cognitive processes that enhanced phonetic recall, which was not found to reflect chunking beyond chance.

It is unlikely that in the present case the PSWs resulting from semantic processing, specifically at Fz and Cz, but not Pz, indexed encoding strategies that enhance recall of single items because of scalp location (e.g., Fabiani et al., 1986, 1990; Fabiani and Donchin, 1995). Additionally, if this were so, the relations between the amplitudes of the PSWs should have been similar whether the three semantic words in the triads were recalled in the same order (three-word chunks) or not. However, regression fit occurred only when recall in three-word chunks was measured, at Fz and Cz. For the phonetically related items, though, it seems that the observed deflections at Cz and Pz may reflect cognitive processes that increase recall of single items (P300) because these words were indeed well-recalled, but not in a chunked manner. Because these deflections were not as pronounced and were prolonged in comparison to the ones resulting from perceptual distinctiveness (7th word in the perceptual list), they may also possibly indicate different cognitive processes, or a different kind of PSW (see below).

The PSWs resulting from the presentation of the semantically related words, which elicited chunking, could have reflected associative processing (Kim et al., 2009, 2012) found in paradigms that involved sequential presentations of pairs of related words. However, our PSWs only partly shared the characteristics of the frontal deflection described by Kim et al. (2009), which occurred much later (began after 1 s of word presentation), and differed from the PSWs found by Kim et al. (2012), which were larger at posterior sites, though they coincided with ours in terms of latency. Both these late positivities of Kim et al.'s studies indexed better recall of associated words, but differed in latency and scalp location in spite of having involved the same paradigm. A possible explanation for these differences between their studies, and also between their studies and ours, is that in their case recall due to chunking may have been confounded with better encoding of single items, that also improve memory. Because they used cued recall, measuring recall of the word that was associated to the other item in the pair does not allow the determination of chunking as conducted here (see the Introduction Section for more details). The contrasts between our and Kim et al.'s (2009, 2012) studies are also possibly due to the use of different methods. For example, they used encoding of lists of paired associates, with manipulations of intra-list semantic similarity only, tested memory using cued-recall, among other differences. In our case, the less predictable nature of the association between stimuli, and the overall list context of unrelated words, likely led to different types of encoding mechanisms depending on the type of association encountered within each type of triad. Because we found that PSW amplitudes at Fz and Cz correlated with “real” semantic three-word chunks, we propose that it is possible to establish the role of PSW (at Fz and Cz) in chunking when processes related to strengthening of individual memory traces and chunking (or “bonding”) are teased apart.

Concerning PSW scalp location, it must also be considered that depending on brain topography, the positive deflections in this latency range can relate to bottom-up/automatic (centro-parietal) or top-down (frontal), effortful, associative or elaborative processes (see Li et al., 2010), all of which yield increases in episodic memory formation (Fernández and Tendolkar, 2001). Chunking of information can occur for both of these types of processes (e.g., Fernández and Tendolkar, 2001; Gobet et al., 2001; Baddeley, 2012; Jaswal, 2012; Kazerounian and Grossberg, 2014), and the formation of relational memories involving parallel and bi-directional interactions between temporal and prefrontal brain areas could be involved in generating these PSWs (Fernández and Tendolkar, 2001). Previous behavioral studies from our lab have, in fact, shown that the facilitation in remembering semantically related words in the paradigm used here can be driven by automatic processes (e.g., see Andrade et al., 2003; Nogueira et al., 2006). Despite this possible automatic linking of verbal information, which may have occurred for phonetically related items, and triggered the PSWs at Cz and Pz, maintaining this activity demands attention/executive/frontal functioning (see Baddeley, 2012; Jaswal, 2012), possibly reflected more frontally. Moreover, this maintenance is essential to allow further manipulation that can lead to better memorization of chunked stimuli (see Fernández and Tendolkar, 2001; Bor et al., 2004; Baddeley, 2012) by interlinking multiple items, a process closely related to fronto-parietal network activation (see Bor and Seth, 2012; Tsuchiya and van Boxtel, 2013). This may have led to larger deflections at Fz, extending to Cz, for semantically related words. Importantly, this type of processing can be distinguished from detection of relatedness (N400) using the present paradigm. It should be kept in mind, however, that the present experiment was not designed to determine the brain regions that were at play in generating the PSWs, for which source analysis would have been essential. Furthermore, it cannot be excluded that physiological changes related to the generation of PSWs, plus those that diminish the negativity of the N400, which also changed progressively as more related words were presented, could have added up to enhanced chunking (see Kim et al., 2012).

Another aspect to consider is that brain changes that result in chunking occur over the period in which the various chunkable items are presented, and possibly after. It is therefore quite difficult to pinpoint just one deflection over a set of chunkable stimuli that correlate very highly with chunking. Consider, for example, the 7th words in the semantic and phonetic lists (the first words in the triads), which were better recalled than the words in the same positions in the control lists. How could this have happened if they were unrelated to the prior word, just as in the control list? In other words, only after the 8th words were presented could participants realize that the 7th word was related to it, so no ERP changes related to chunking could be visible during the presentation of the 7th words. What seems to have occurred is that in the semantic lists the 7th word was integrated with the other words of the triad to form a chunk *backwards* in time in relation to the ERP signal collected while participants saw the 7th word. Thus, cognitive processing that facilitated recall must have happened *after* the presentations of the 7th words. Our data provide a good indication that this occurs during the presentation of the 8th and 9th words (second and third related word in the triads). This may, however, still have continued during the presentation of the 10th word, because PSWs measured during the presentation of this word were still more positive at Fz in the semantic lists than the control and perceptual lists. Additionally, during the presentation of the 8th and 9th words, and even the 10th, many different processes could have occurred, such as strengthening individual memory traces of each item individually, as well as binding of any of the words in the triad with one or two of the others. Hence, the PSWs observed for the 9th words could only partially, or indirectly, account for the association between this deflection and chunking of the three words in the semantic triads. This may explain why the correlations we found were not higher. Note that the analysis of subsequent memory effects (see Picton et al., 2000) would also not succeed in explaining our findings because the ERP changes for each word in the triad does not reflect their individual recall: when chunking occurs, recall of one item brings the others to mind. Still, our objective was to show whether the paradigm used here would be promising in dissociating processes related to detection of distinctiveness, associative processes and other mechanisms related to chunking. In this we believe we were successful. Furthermore, our paradigm allows the study of encoding and immediate free recall of stimuli without the confounding of mechanisms related to primacy and recency effects (see Kahana, 1996; Nogueira et al., 2006), while the use of three related words also takes into consideration the number of items that can be stored in working memory at the same time (4 ± 1; see Cowan, 2010; Kazerounian and Grossberg, 2014).

A possible solution to understanding how PSWs relate to chunking is to consider this deflection in terms of the computational models of chunking discussed by Kazerounian and Grossberg (2014). The fact that these deflections increased as more semantic items in the triad were presented indicates that they cannot have indexed activation related to Item-and-Order processes to individual stimuli, which would have led to a decrease in activation. This is so because Item-and-Order activity to individual stimuli responds to a primacy gradient (i.e., activity related to each item in a sequence decreases progressively; see Kazerounian and Grossberg, 2014). However, these progressively larger PSWs could reflect Masking Fields, which respond to sequences of chunkable items and have greater interaction strengths for chunks with more items. This occurs either because of larger recurrent *inhibitory* activity and/or stronger top-down *excitatory* signals of larger chunks over smaller ones (see Kazerounian and Grossberg, 2014). Which of these signals, inhibitory or excitatory, are related to the PSWs at Fz and Cz deserve further investigations.

In summary, we showed that the presentation of three sequentially presented words that are related with each other in different ways, and distinct from their context, has the potential of uncovering ERP components that have been proposed as measures of chunking (PSW) that can be dissociated from classic P300- and N400-like deflections. PSWs became apparent when semantic associations were possible, having been more pronounced when more items could be associated. Furthermore, the amplitude of these waves was correlated with chunking of three successive semantically related items. We therefore presented a methodological advance in providing a paradigm which shows cognitive processes involved in chunking that are distinct from other mechanisms that increase immediate free recall. Notwithstanding, this study must be followed by investigations that use appropriate techniques to detect the brain regions that originated the ERP signals, as well as the electrophysiological alterations that occur when people retrieved these items. Contrasting our findings with predictions of recall patterns from computational models of chunking may also be an productive approach.

In contrast to the main strength of the present work, which took into consideration recent models of memory in the construction of the paradigm, as well as the detailed behavioral analysis of the data, our study is limited by the use of few electrodes. This was due to technical constraints of our equipment, which did not allow us to carry out source or coherence analysis to determine the brain regions involved in the studied deflections. These, however, were not the objectives of our investigation. Note that the different encoding instructions to those used here may lead to different effects (see Nelson et al., 1987; Weyerts et al., 1997) and that our behavioral and electrophysiological findings are specific to the population under investigation (young, healthy individuals in optimum cognitive conditions).

### Conflict of interest statement

The authors declare that the research was conducted in the absence of any commercial or financial relationships that could be construed as a potential conflict of interest.
